# Supplementary material for: Cortical tau deposition follows patterns of entorhinal functional connectivity in aging
Source: eLife. 2019 Sep 2;8:e49132. doi: 10.7554/eLife.49132 (PMC6764824; doi:10.7554/eLife.49132)
Supplement: Supplementary file 1. [file elife-49132-supp1.docx]

**Supplementary file 1.** Results of ANCOVA models investigating tau deposition within regions of functional connectivity from the older adult sample.

| ANCOVA 1: EC FC vs. Outside Cortical Regions (OA FC) | | | |
| --- | --- | --- | --- |
| Within Subjects Effects | *df* | *F* | *p* |
| Region | 1 | 115.93 | <0.001 |
| Region x Aβ status | 1 | 12.34 | 0.001 |
| Post-hoc paired t-tests | *df* | *t* | *p* |
| FTP in EC FC vs. outside | 121 | 9.73 | <0.001 |
| Post-hoc independent samples t-tests | *df* | *t* | *p* |
| FTP mean difference in Aβ+ vs. Aβ- | 69.40 | 3.25 | 0.002 |
| ANCOVA 2: alEC FC vs. pmEC FC vs. Outside Cortical Regions (OA FC) | | | |
| Within Subjects Effects | *df* | *F* | *p* |
| Region | 1.86 | 36.55 | <0.001 |
| Region x Aβ status | 1.86 | 2.84 | 0.07 |
| Post-hoc paired t-tests | *df* | *t* | *p* |
| FTP in alEC FC vs. outside | 121 | 7.09 | <0.001 |
| FTP in pmEC FC vs. outside | 121 | 6.75 | <0.001 |
| FTP in alEC FC vs. pmEC FC | 121 | 2.11 | 0.04 |

Repeated measures ANCOVA models comparing tau deposition, or the proportion of suprathreshold FTP voxels (>1.4 SUVR), in each region (Model 1: EC FC vs. Outside Cortical Regions; Model 2: alEC FC vs. pmEC FC vs. Outside Cortical Regions) as a within-subjects factor, Aβ status as a between subjects factor, and age and sex as covariates of no interest. Significant main effects of region were analyzed post-hoc with paired samples t-tests comparing tau deposition between regions. Significant region by Aβ status interactions were analyzed post-hoc with independent samples t-tests, comparing the mean difference in tau deposition between regions across Aβ+ and Aβ- groups. FC, functional connectivity; EC, entorhinal cortex; alEC, anterolateral EC; pmEC, posteromedial EC; OA, older adult.
