# Supplementary material for: Cortical tau deposition follows patterns of entorhinal functional connectivity in aging
Source: eLife. 2019 Sep 2;8:e49132. doi: 10.7554/eLife.49132 (PMC6764824; doi:10.7554/eLife.49132)
Supplement: Supplementary file 2. [file elife-49132-supp2.docx]

**Supplementary file 2.** Associations between functional connectivity specific tau deposition and both Aβ and EC tau.

| FC Specific Tau Deposition | Global PiB DVR | | FC Mask  PiB DVR | | Mean EC  FTP SUVR | | EC FTP cont. for Global FTP | |
| --- | --- | --- | --- | --- | --- | --- | --- | --- |
|  | r | p | r | p | r | p | r | p |
| YA FC Masks (correlations across all OA participants) | | | | | | | | |
| EC FC | 0.37 | <0.001 | 0.38 | <0.001 | 0.62 | <0.001 | 0.51 | <0.001 |
| alEC FC | 0.47 | <0.001 | 0.47 | <0.001 | 0.46 | <0.001 | 0.23 | 0.01 |
| pmEC FC | 0.20 | 0.03 | 0.23 | 0.01 | 0.30 | 0.001 | 0.05 | 0.59 |
| alEC-pmEC FC | 0.41 | <0.001 | - | - | 0.33 | <0.001 | 0.21 | 0.02 |
| YA FC Masks (correlations within Aβ+ participants only) | | | | | | | | |
| EC FC | 0.30 | 0.04 | 0.34 | 0.02 | - | - | - | - |
| alEC FC | 0.44 | 0.002 | 0.45 | 0.002 | - | - | - | - |
| pmEC FC | 0.17 | 0.26 | 0.23 | 0.12 | - | - | - | - |
| alEC-pmEC FC | 0.40 | 0.005 | - | - | - | - | - | - |
| OA FC Masks (correlations across all OA participants) | | | | | | | | |
| EC FC | 0.41 | <0.001 | 0.43 | <0.001 | 0.54 | <0.001 | 0.33 | <0.001 |
| alEC FC | 0.33 | <0.001 | 0.37 | <0.001 | 0.38 | <0.001 | 0.14 | 0.12 |
| pmEC FC | 0.20 | 0.03 | 0.24 | 0.01 | 0.31 | 0.001 | -0.07 | 0.43 |
| alEC-pmEC FC | 0.19 | 0.04 | - | - | 0.15 | 0.11 | 0.19 | 0.04 |
| OA FC Masks (correlations within Aβ+ participants only) | | | | | | | | |
| EC FC | 0.34 | 0.02 | 0.38 | 0.008 | - | - | - | - |
| alEC FC | 0.31 | 0.04 | 0.37 | 0.01 | - | - | - | - |
| pmEC FC | 0.25 | 0.10 | 0.29 | 0.05 | - | - | - | - |
| alEC-pmEC FC | 0.15 | 0.33 | - | - | - | - | - | - |

All correlations are Pearson’s partial correlations controlling for age and sex (and additional variables if specified). FC, Functional connectivity; FC specific tau deposition was defined as the mean difference in the proportion of suprathreshold FTP voxels (>1.4 SUVR) between regions of FC and outside cortical regions, or the mean difference between regions of alEC FC and pmEC FC; YA, young adult; OA, older adult; mean EC FTP SUVR, the mean partial volume FTP signal within a FreeSurfer derived EC ROI.
