# Supplementary material for: Cortical tau deposition follows patterns of entorhinal functional connectivity in aging
Source: eLife. 2019 Sep 2;8:e49132. doi: 10.7554/eLife.49132 (PMC6764824; doi:10.7554/eLife.49132)
Supplement: Supplementary file 3. [file elife-49132-supp3.docx]

**Supplementary file 3.** Results of ANCOVA models comparing tau deposition in different functional connectivity strength regions from the older adult sample.

| ANCOVA 1: EC FC Strength (OA FC) | | | |
| --- | --- | --- | --- |
| Within Subjects Effects | *df* | *F* | *p* |
| FC Strength | 1.16 | 71.81 | <0.001 |
| FC Strength x Aβ status | 1.16 | 13.67 | <0.001 |
| Post-hoc paired t-tests | *df* | *t* | *p* |
| FTP in medium vs. low FC | 121 | 5.86 | <0.001 |
| FTP in high vs. medium FC | 121 | 7.32 | <0.001 |
| FTP in high vs. low FC | 121 | 7.71 | <0.001 |
| Post-hoc ind. samples t-tests (Aβ+ vs. Aβ-) | *df* | *t* | *p* |
| Mean difference in medium vs. low FC | 64.75 | 2.44 | 0.02 |
| Mean difference in high vs. medium FC | 69.12 | 3.51 | 0.001 |
| Mean difference in high vs. low FC | 72.40 | 3.42 | 0.001 |
| ANCOVA 2: alEC FC Strength (OA FC) | | | |
| Within Subjects Effects | *df* | *F* | *p* |
| FC Strength | 1.06 | 15.00 | <0.001 |
| FC Strength x Aβ status | 1.06 | 4.61 | 0.03 |
| Post-hoc paired t-tests | *df* | *t* | *p* |
| FTP in medium vs. low FC | 121 | 0.02 | 0.99 |
| FTP in high vs. medium FC | 121 | 3.72 | <0.001 |
| FTP in high vs. low FC | 121 | 3.32 | 0.001 |
| Post-hoc ind. samples t-tests (Aβ+ vs. Aβ-) | *df* | *t* | *p* |
| Mean difference in medium vs. low FC | 59.71 | 0.82 | 0.41 |
| Mean difference in high vs. medium FC | 77.58 | 2.08 | 0.04 |
| Mean difference in high vs. low FC | 80.04 | 2.14 | 0.04 |
| ANCOVA 3: pmEC FC Strength (OA FC) |  |  |  |
| Within Subjects Effects | *df* | *F* | *p* |
| FC Strength | 1.32 | 32.07 | <0.001 |
| FC Strength x Aβ status | 1.32 | 2.68 | 0.09 |
| Post-hoc paired t-tests | *df* | *t* | *p* |
| FTP in medium vs. low FC | 121 | -7.64 | <0.001 |
| FTP in high vs. medium FC | 121 | -1.93 | 0.06 |
| FTP in high vs. low FC | 121 | -5.76 | <0.001 |

Repeated measures ANCOVA models compared tau deposition, or the proportion of suprathreshold FTP voxels (>1.4 SUVR), in each FC strength region (low vs. medium vs. high FC) as a within-subjects factor, Aβ status as a between subjects factor, and age and sex as covariates of no interest (Model 1: EC FC; Model 2: alEC FC; Model 3: pmEC FC). Significant main effects of FC strength were analyzed post-hoc with paired samples t-tests comparing tau deposition between FC strength regions. Significant region by Aβ status interactions were analyzed post-hoc with independent samples t-tests, comparing the mean difference in tau deposition between FC strength regions across Aβ+ and Aβ- groups. FC, functional connectivity; EC, entorhinal cortex; alEC, anterolateral EC; pmEC, posteromedial EC; OA, older adult.
