# Supplementary material for: Cortical tau deposition follows patterns of entorhinal functional connectivity in aging
Source: eLife. 2019 Sep 2;8:e49132. doi: 10.7554/eLife.49132 (PMC6764824; doi:10.7554/eLife.49132)
Supplement: Supplementary file 4. [file elife-49132-supp4.docx]

**Supplementary file 4.** Quantification of voxels retained within seed regions after removing signal drop out.

| Seed | YA Mean (Range) | OA Mean (Range) |
| --- | --- | --- |
| EC-L | 98.9% (93.0-100%) | 98.3% (90.7-100%) |
| EC-R | 99.7% (95.2-100%) | 98.7% (92.2-100%) |
| alEC-L | 98.9% (93.6-100%) | 99.3% (94.7-100%) |
| alEC-R | 99.6% (95.6-100%) | 99.4% (92.7-100%) |
| pmEC-L | 96.8% (89.5-100%) | 97.8% (92.1-100%) |
| pmEC-R | 96.8% (90.6-100%) | 97.5% (90.6-100%) |

Voxels with a mean intensity of <2 SD from the mean intensity across voxels within the seed were removed from each seed region on a participant level basis (See Methods). Mean and range of the percentage of voxels retained in the Young Adult (YA) and OA (OA) samples are presented.
