# Supplementary material for: Cortical tau deposition follows patterns of entorhinal functional connectivity in aging
Source: eLife. 2019 Sep 2;8:e49132. doi: 10.7554/eLife.49132 (PMC6764824; doi:10.7554/eLife.49132)
Supplement: Transparent reporting form [file elife-49132-transrepform.docx]

***eLife’s* transparent reporting form**

We encourage authors to provide detailed information *within their submission* to facilitate the interpretation and replication of experiments. Authors can upload supporting documentation to indicate the use of appropriate reporting guidelines for health-related research (see [EQUATOR Network](http://www.equator-network.org/%20)), life science research (see the [BioSharing Information Resource](https://biosharing.org/)), or the [ARRIVE guidelines](http://www.plosbiology.org/article/info:doi/10.1371/journal.pbio.1000412) for reporting work involving animal research. Where applicable, authors should refer to any relevant reporting standards documents in this form.

If you have any questions, please consult our Journal Policies and/or contact us: [editorial@elifesciences.org](mailto:editorial@elifesciences.org).

**Sample-size estimation**

- You should state whether an appropriate sample size was computed when the study was being designed
- You should state the statistical method of sample size computation and any required assumptions
- If no explicit power analysis was used, you should describe how you decided what sample (replicate) size (number) to use

Please outline where this information can be found within the submission (e.g., sections or figure legends), or explain why this information doesn’t apply to your submission:

No explicit power analysis was conducted to determine sample size. All usable data from our larger cohort study (BACS) was included in the current study. Inclusion criteria for BACS is described in *Methods*. Final sample sizes for each element of the study (fMRI, PET) is detailed in *Methods* and *Table 1*, and these samples are of similar sizes to previous studies using fMRI and PET.

**Replicates**

- You should report how often each experiment was performed
- You should include a definition of biological versus technical replication
- The data obtained should be provided and sufficient information should be provided to indicate the number of independent biological and/or technical replicates
- If you encountered any outliers, you should describe how these were handled
- Criteria for exclusion/inclusion of data should be clearly stated
- High-throughput sequence data should be uploaded before submission, with a private link for reviewers provided (these are available from both GEO and ArrayExpress)

Please outline where this information can be found within the submission (e.g., sections or figure legends), or explain why this information doesn’t apply to your submission:

No replications were performed in this study. For the fMRI data, outlier volumes with high motion were identified and their effects were removed from the data as described in *Methods, “*fMRI Preprocessing*”*. No outlier removal was performed for the PET data.

**Statistical reporting**

- Statistical analysis methods should be described and justified
- Raw data should be presented in figures whenever informative to do so (typically when N per group is less than 10)
- For each experiment, you should identify the statistical tests used, exact values of N, definitions of center, methods of multiple test correction, and dispersion and precision measures (e.g., mean, median, SD, SEM, confidence intervals; and, for the major substantive results, a measure of effect size (e.g., Pearson's r, Cohen's d)
- Report exact p-values wherever possible alongside the summary statistics and 95% confidence intervals. These should be reported for all key questions and not only when the p-value is less than 0.05.

Please outline where this information can be found within the submission (e.g., sections or figure legends), or explain why this information doesn’t apply to your submission:

All statistical methods are described in detail in *Methods*, and concise descriptions are provided in the *Results* section associated with each analysis. Exact values of N and dispersion and precision measures are reported for each statistical analysis in *Results.* Corrections for multiple comparisons are described in *Methods* for both fMRI analyses (“Functional Connectivity Analysis” – “Second level analysis” subsection) and all other statistical tests (“Statistical Analysis”). We report exact p-values for values >0.001 in the *Results* section for all primary analyses.

(For large datasets, or papers with a very large number of statistical tests, you may upload a single table file with tests, Ns, etc., with reference to sections in the manuscript.)

**Group allocation**

- Indicate how samples were allocated into experimental groups (in the case of clinical studies, please specify allocation to treatment method); if randomization was used, please also state if restricted randomization was applied
- Indicate if masking was used during group allocation, data collection and/or data analysis

Please outline where this information can be found within the submission (e.g., sections or figure legends), or explain why this information doesn’t apply to your submission:

N/A

**Additional data files (“source data”)**

- We encourage you to upload relevant additional data files, such as numerical data that are represented as a graph in a figure, or as a summary table
- Where provided, these should be in the most useful format, and they can be uploaded as “Source data” files linked to a main figure or table
- Include model definition files including the full list of parameters used
- Include code used for data analysis (e.g., R, MatLab)
- Avoid stating that data files are “available upon request”

Please indicate the figures or tables for which source data files have been provided:

Numerical data has been provided for main results (includes Table 1, Figure 2, Figure 3, Figure 2-Figure Supplement 1, Figure 3-Figure Supplement 1, Supplementary File 1, Supplementary File 2), and are named as “Source Data” with the corresponding table/figure. We have not included data from figures based upon images (such as the fMRI data in Figure 1).

Data analysis was performed with standard codes and analysis packages that are publically available (such as CONN, SPSS, and SPM), and these codes/packages have been listed in *Methods* in association with each analysis.
